# Postural Control in Bilateral Vestibular Failure: Its Relation to Visual, Proprioceptive, Vestibular, and Cognitive Input

**DOI:** 10.3389/fneur.2017.00444

**Published:** 2017-09-01

**Authors:** Andreas Sprenger, Jann F. Wojak, Nico M. Jandl, Christoph Helmchen

**Affiliations:** ^1^Department of Neurology, University of Lübeck, Lubeck, Germany; ^2^Institute of Psychology II, University of Lübeck, Lubeck, Germany

**Keywords:** bilateral vestibular failure, postural control, posturography, proprioception, multisensory integration

## Abstract

Patients with bilateral vestibular failure (BVF) suffer from postural and gait unsteadiness with an increased risk of falls. The aim of this study was to elucidate the differential role of otolith, semicircular canal (SSC), visual, proprioceptive, and cognitive influences on the postural stability of BVF patients. Center-of-pressure displacements were recorded by posturography under six conditions: target visibility; tonic head positions in the pitch plane; horizontal head shaking; sensory deprivation; dual task; and tandem stance. Between-group analysis revealed larger postural sway in BVF patients on eye closure; but with the eyes open, BVF did not differ from healthy controls (HCs). Head tilts and horizontal head shaking increased sway but did not differ between groups. In the dual task condition, BVF patients maintained posture indistinguishable from controls. On foam and tandem stance, postural sway was larger in BVF, even with the eyes open. The best predictor for the severity of bilateral vestibulopathy was standing on foam with eyes closed. Postural control of our BVF was indistinguishable from HCs once visual and proprioceptive feedback is provided. This distinguishes them from patients with vestibulo-cerebellar disorders or functional dizziness. It confirms previous reports and explains that postural unsteadiness of BVF patients can be missed easily if not examined by conditions of visual and/or proprioceptive deprivation. In fact, the best predictor for vestibular hypofunction (VOR gain) was examining patients standing on foam with the eyes closed. Postural sway in that condition increased with the severity of vestibular impairment but not with disease duration. In the absence of visual control, impaired otolith input destabilizes BVF with head retroflexion. Stimulating deficient SSC does not distinguish patients from controls possibly reflecting a shift of intersensory weighing toward proprioceptive-guided postural control. Accordingly, proprioceptive deprivation heavily destabilizes BVF, even when visual control is provided.

## Introduction

Bilateral vestibular failure (BVF) is characterized by unsteadiness of stance and gait and disabling oscillopsia during head movements (1). BVF has a wide spectrum of etiologies (2, 3), ranging from vestibulo-toxic agents such as antibiotics (4, 5), opioids (6), salicyl acid (7), amiodarone (8) and chemotherapy (9, 10); and polyneuropathies (11–13) to sequential vestibulopathies, e.g., due to Menière’s disease or vestibular neuritis. Most often BVF remains idiopathic. Rarer causes include systemic autoimmune diseases, e.g., Cogan’s syndrome (14), in particular connective tissue disease, e.g., systemic lupus erythematosus, Behcet’s disease, neurosarcoidosis but also infectious diseases (e.g., borreliosis), vitamine B1 deficiency (15), schwannoma, meningeosis, superficial siderosis (16) and it may present as part of neurodegenerative diseases, e.g., idiopathic cerebellar ataxia with BVF (17, 18) and additional polyneuropathy CANVAS syndrome (19). In line with the variety of etiologies, vestibular hypofunction may encompass semicircular canal (SSC) and otolith signal processing in the labyrinth or vestibular nerve separately or combined. Moderate vestibular hypofunction may also come from cerebellar disease (20) which also causes postural unsteadiness.

Postural ataxia in peripheral BVF may be related to abnormal otolith processing and/or SSC malfunction in the inferior and superior branch of the vestibular nerve or within the labyrinth (21). Since the SSC senses rotatory head acceleration patients might complain about dizziness and unsteadiness particularly on head and body rotations, whereas patients with abnormal otolith function might rather complain about dizziness on linear acceleration or tilted head positions. Using foam posturography postural ataxia increased with the severity of combined otolith and SSC hypofunction (22, 23). Vestibular hypofunction may be compensated by substitution by other sensory systems and/or central compensation (24). A few lines of behavioral and brain imaging (25) evidence indicate a change in intersensory weighing to compensate for postural ataxia (26). One example for a shift of sensory weighing is the increased visual dependence during transient vestibular loss in weightlessness [e.g., micro-gravity, spacelab (27, 28)]. Therefore, we hypothesized that BVF patients show increased sensitivity to proprioceptive and visual input. However, it is unknown how patients with partial, i.e., incomplete lesions of the vestibular afferents stabilize stance when vestibular otolith or SSC stimuli are applied during postural control. Our primary aim was to compare postural control in BVF and healthy control (HC) subjects by systematically modulating visual, SSC, otolith, and proprioceptive input. As postural control might be influenced by focused attention and/or cognitive distraction [dual task (29)] and more challenging balance tasks (tandem stance), we added these conditions to elaborate how these factors might unmask latent postural instability in BVF.

## Materials and Methods

### Participants

Patients were diagnosed to have BVF based on clinical examinations, bithermal caloric irrigation [bilateral hyporesponsiveness with mean peak slow phase velocity (SPV) of <5°/s on both sides], and quantitative head impulse recordings of the vestibulo-ocular reflex (VOR, reduced gain <0.7), absence of clinical signs for cerebellar disease, and normal cranial MRI. On clinical examination, all patients showed gait ataxia without significant consistency in lateropulsion/gait deviation. Gait ataxia severely increased with horizontal head movements while attempting to fixate targets at gaze straight ahead. Romberg’s test was pathological in all of them while the Unterberger test was not pathological (no consistent deviation) in any of the patients. A total number of 31 patients with chronic (>3 months, range: 3 months to 20 years) BVF were examined (mean VOR gain: 0.26). Nine patients had to be excluded due to comorbidity (polyneuropathy). This resulted in 22 eligible BVF patients [12 male; age: 64.0 ± 2.2 years (SE); disease duration: range 3 months to 20 years; mean 3.1 years]. The most common etiology of BVF was antibiotic ototoxicity (*n* = 13), unknown cause (*n* = 8), and sequential vestibular neuritis (*n* = 1). The patient and the HC group (*n* = 28, 17 male; age: 65.2 ± 1.7 years; mean gain 0.97 ± 0.02) did not differ significantly in age (two-sample *t*-test *p* = 0.68), gender (chi-square test *p* = 0.77), or Montreal Cognitive Assessment test score (two-sample *t*-test *p* = 0.52) [MoCA (30)].

### Electrophysiological and Psychophysical Recordings

Semicircular canal function was investigated by electronystagmography with caloric irrigation and quantitative head impulse testing; otolith function by static (background stationary) and dynamic (moving visual background) subjective visual vertical (SVV) (31) and cervical and ocular vestibular-evoked myogenic potentials [VEMP (32)]. Cervical VEMP were elicited by asking the lying participants to slightly lift their heads and maintain a tonic rotational position of their heads to the contralateral side while EMG activity was recorded from the mid portion of the sternocleidomastoid muscles. Unilateral AC tone bursts of 500 Hz were used and p13-n23 components were analyzed [for details see Ref. (32, 33)].

All participants were examined by quantitative head impulse test using video-oculography. Eye and head movements were recorded by the EyeSeeCam^®^ HIT System (Autronics, Hamburg, Germany) at a sampling rate of 220 Hz (34). VOR gain was determined by robust linear regression of eye and head velocity starting at head velocity >10°/s to 95% of peak head velocity using Matlab^®^(The Mathworks Inc., Natick, MA, USA, version R2016a). For further details, see Ref. (35–37).

### Experimental Conditions

**Figure 1:**
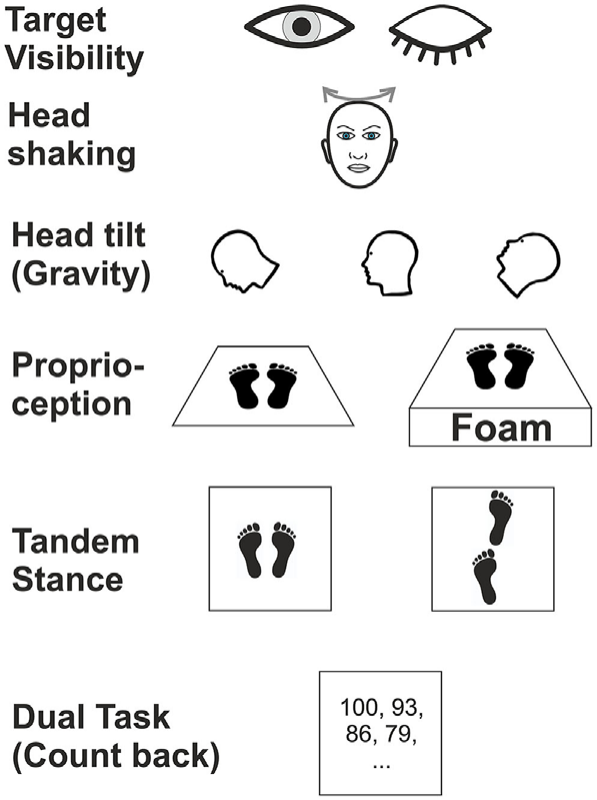
Schematic illustration of six experimental conditions in which subjects were examined during posturography.

Posturography was recorded in the upright standing position with the hands hanging next to the trunk for 20 s. At baseline, subjects were asked to stand on the platform with feet (shoes) parallel to each other. We tested various experimental conditions (Figure 1) which differed in terms of (i) visual (eyes open/closed; EO/EC), (ii) graviceptive otolith (head tilted up, down vs. head erect, with the eyes open and closed) (38), (iii) SSC (horizontal head shaking) (iv) and proprioceptive (foam) input, (v) cognitive influence (dual task with backward counting), and (vi) complex motor challenging demands on postural control (tandem stance) (39). During horizontal head shaking (0.5 Hz) participants were asked to fixate a target 60 cm in front of the participants’ forehead. Head movements were recorded and monitored with the ZEBRIS system (CMS70P, Zebris Medizintechnik GmbH, Isny, Germany) at a sampling rate of 50 Hz (40). The system determines the position [specified by three values *v* = (*x, y, z*)] of an ultrasound-emitting marker relative to an array of three receivers. This condition was meant to compare the effects of vestibular semicircular canal stimulation on vestibulo-spinal postural control in patients with incomplete lesions of the vestibulo-ocular reflex, with and without visual feedback (eyes open vs. closed). Although this technique is not a selective stimulation of the horizontal canals, the effects on the horizontal SSC are expected to be much stronger than on the vertical SSC and on the utricles.

Head position was adjusted by an inclinometer (38). This recording assured that the different head positions (anteflection by 45°, upright head positions, 30° dorsoflection of the neck; with gaze straight ahead relative to head position) were maintained for the recording time. We used a slab of foam rubber (50 cm width, 60 cm length, height 10 cm, compression hardness: 3.3 kPa, volumetric weight: 40 kg/m^3^) for testing balance control under attenuated proprioceptive feedback under two conditions: (a) with the head erect, gaze fixation of LED at the gaze straight ahead position and (b) with the eyes closed.

### Posturography

We used a Kistler force platform (Model 9260AA6, Kistler Instrumente AG, Winterthur, Switzerland; 50 cm width, 60 cm length, height 10 cm) equipped with piezo-electric 3-component force sensors for recording postural changes during the above mentioned experimental conditions in a similar way as described elsewhere (41, 42). Postural sway signals were bidirectionally filtered (50 Hz Gaussian filter) to eliminate low amplitude recording noise (43). The platform recorded torques and sheer forces with six degrees of freedom using force transducers with an accuracy better than 0.5 N. The displacement of the center of pressure in the medio-lateral (ML) and the anterior–posterior (AP) directions were recorded and the sum vector calculated using Matlab^®^. Results are given as the mean postural sway speed (PSS, in centimeter per second), calculated from the AP and ML movements:

$$\text{PSS}=\text{mean}(\sqrt{{\left({\text{AP}}_{i}-{\text{AP}}_{i-1}\right)}^{2}+{\left({\text{ML}}_{i}-{\text{ML}}_{i-1}\right)}^{2}}*\text{SamplingRate})$$

Postural sway was recorded in intervals of 20 s duration for off-line analysis (sampling frequency 250 Hz) (39).

### Statistical Analysis

Statistical analyses were performed with SPSS (22.0.0.2; IBM Corp., Somer, NY, USA). Analyzing the postural sway speed, the factors TARGET VISIBILITIY (eyes open/closed), HEAD POSITION, HEAD SHAKING, DUAL TASK (counting), PROPRIOCEPTION (foam), and TANDEM STANCE were taken as within-subject factors and group as between-subjects factor. Analyzing Romberg’s ratio the factor TARGET VISIBILITY was eliminated, therefore all other factors were included in the ANOVA. In some comparisons sphericity requirement was violated. Therefore, we report *F*-values with Greenhouse-Geisser correction but report degrees of freedom (df) uncorrected in order to show the factorial analysis design. Statistical comparisons were performed parametric unless stated otherwise.

Multi-factorial ANOVA with the above mentioned factors were performed. Significance levels of these tests were Bonferroni corrected for multiple testing. Statistical differences were regarded as significant for values *p* < 0.05. Error bars indicate SEM. Correlation analyses were performed using Spearman-Rho coefficient unless otherwise stated. The effects of visual deprivation on postural stability were determined by Romberg’s ratio computing PSS with the eyes closed/eyes open (22).

## Results

### Electrophysiological Data

The mean VOR gain was reduced to 0.26 ± 0.04 indicating severe bilateral vestibulopathy. Mean peak SPV of caloric nystagmus was 4.5 ± 0.8°/s. oVEMP were recorded in 22 patients and 23 HC subjects; they were absent in 12 patients and revealed reduced amplitudes in the other 10 patients: peak amplitude differed significantly between groups (Mann–Withney *U* test, *p* = 0.003, median patients: 3.8 µV, median HC subjects: 6.95 µV). cVEMP were recorded in 22 patients and 23 HC subjects (median: 24.4 µV); they were absent in 17 patients and showed significantly reduced amplitudes in the other five patients (median 8.0 µV; *p* = 0.028). SVV did not show pathological tilts (>2.5°) and did not differ between patients and controls, neither during dynamic nor static SVV.

### Postural Data

Generally, postural sway speed differed between paradigms (*F*(13,36) = 71.716, *p* = 0.001) and revealed an interaction of CONDITION × GROUP (*F*(13,36) = 2.559, *p* = 0.038). ANOVA showed a significant group difference (*F*(1,48) = 7.596, *p* = 0.008), i.e., BVF patients (*n* = 22) showed on average larger PSS than HC participants (*n* = 28).

### Target Visibility

There was a main effect for GROUP (*F*(1,48) = 6.08; *p* = 0.015) and TARGET VISIBILITY (Eyes open/eyes closed; EO/EC) (*F*(1,48) = 73.85; *p* < 0.001), i.e., PSS in patients and controls (solid platform, parallel feet, head upright) was significantly larger during eye closure than during eyes open. With the eyes open, the between-group analysis of PSS, however, did not reveal differences between both groups (*p* = 0.295). There was a significant interaction for TARGET VISIBILITY × GROUP (*F*(1,48) = 6.35; *p* = 0.015), i.e., PSS increased on eye closure more in patients than in controls. The difference for Romberg’s ratio (PSS ratio of EC/EO) between both groups (patients: 3.40 ± 0.0.44; controls: 2.49 ± 0.16) failed to reach significance level (*T*(48) = 1.96; *p* = 0.061). In short, Romberg’s ratio at baseline standing condition was larger in BVF.

### Head Position

An ANOVA on the PSS with the within-subject factors HEAD POSITION and TARGET VISIBILITY and the between-subject factor GROUP revealed main effects for GROUP (*F*(1,48) = 6.070, *p* = 0.017), TARGET VISIBILITY (*F*(1,48) = 67.340, *p* < 0.001), HEAD POSITION (*F*(2,47) = 6.086, *p* = 0.004), and an interaction of TARGET VISIBILITY × GROUP (*F*(1,48) = 6.635, *p* = 0.013) but no interaction of HEAD POSITION × GROUP (*F*(1,48) = 2.161, *p* = 0.124) or HEAD POSITION × TARGET VISIBILITY (*F*(2,47) = 2.974, *p* = 0.061) and no triple interaction (*p* > 0.9).

**Figure 2:**
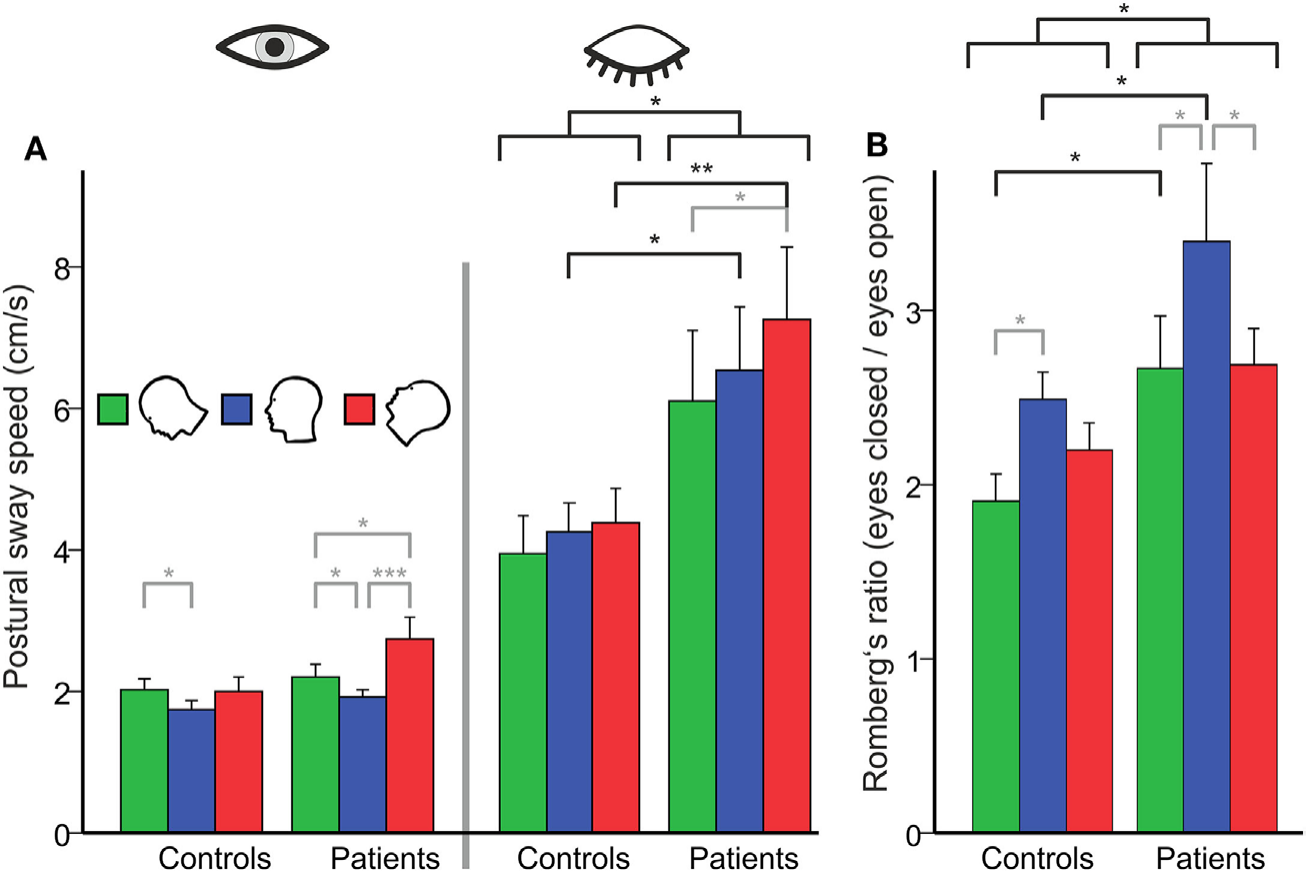
**(A)** Head tilt (gravity)-related effects on postural control in subjects with (left side) and without (right) visual feedback and their relation [Romberg’s ratio **(B)**]. **(A)** Using visual control there is no group difference in postural sway on the firm platform (PSS in centimeter per second). However, BVF patients show significant increases in PSS (left side) in the absence of visual control and during additional gravity effects (head tilt). **(B)** There is a significant higher Romberg’s ratio (right) compared to controls, in contrast to other experimental conditions (dual task, head shaking). Error bars indicate SD; gray lines indicate within-subject differences, black lines indicate between-subject differences; *p < 0.05, **p < 0.01, ***p < 0.001.

A separate ANOVA on PSS with the eyes open revealed no main effect of GROUP but a main effect of HEAD POSITION (*F*(2,47) = 9.845, *p* = 0.001): PSS increased in the head down (nose down) (*p* < 0.001) and head up (nose up) position (*p* = 0.001) with no difference between the gravity-dependent (up vs. down) head positions (Figure 2A). With the eyes closed, there was a main effect for GROUP (*F*(1,48) = 6.453, *p* = 0.014), HEAD POSITION (*F*(2,47) = 3.821, *p* = 0.027) but no interaction HEAD POSITION × GROUP (*p* > 0.4). Analyzing Romberg’s ratio (Figure 2B) there were main effects of GROUP (*F*(1,48) = 6.748, *p* = 0.012) and HEAD POSITION (*F*(2,47) = 7.758; *p* = 0.001) but no interaction of HEAD POSITION × GROUP (*F*(2,48) = 0.793; *p* > 0.45). In BVF patients, Romberg’s ratio was lower in the head up position (*p* = 0.033) and the head down position (*p* = 0.017). In HC, Romberg’s ratio was lower in the head down (*p* = 0.039) but not the head up position. Thus, gravity-dependent tonic head positions in the pitch plane increased postural sway in both groups (no interaction of HEAD POSITION × GROUP) but the increase in postural sway was larger in BVF on eye closure.

### Head Shaking

**Figure 3:**
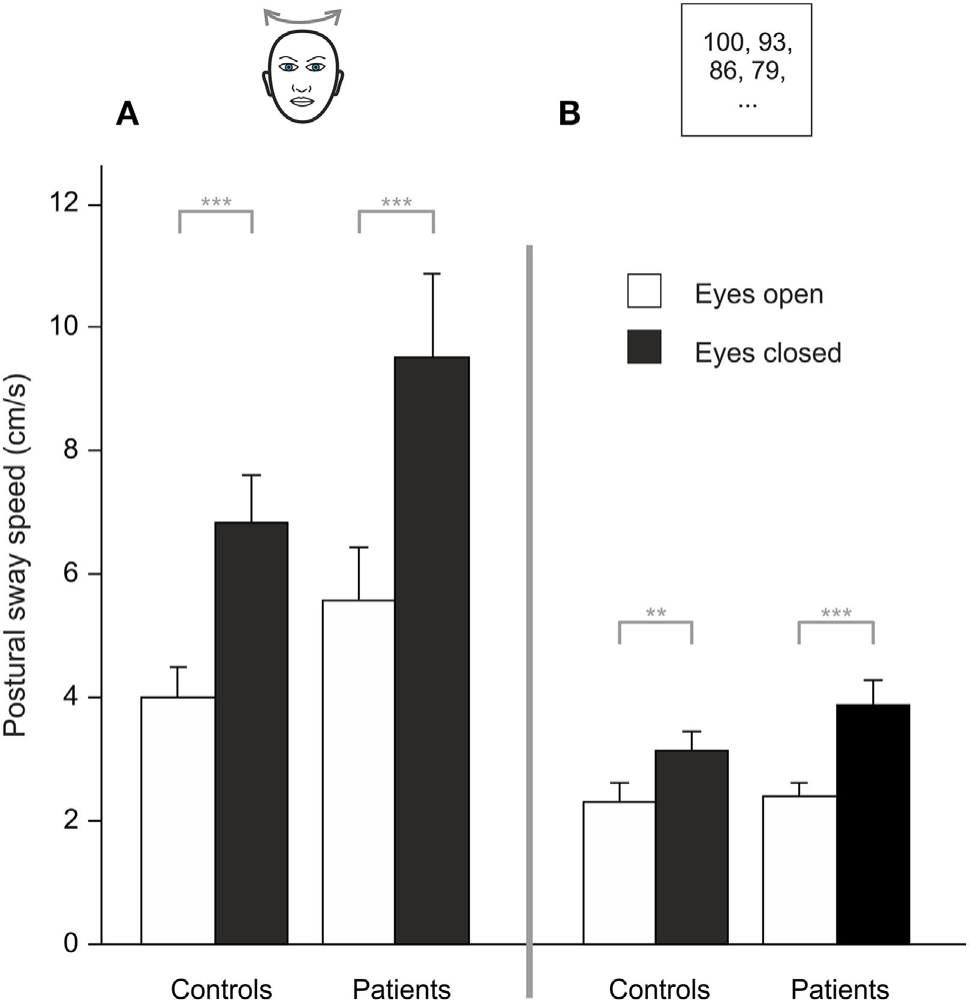
During head shaking **(A)** and dual task **(B)**, patients did not differ from healthy participants but visual information (eyes open/eyes closed) improves postural stability; gray lines indicate within-subject differences; **p < 0.01, ***p < 0.001.

Analyzing PSS during HEAD SHAKING there was a trend for a main effect of GROUP (*F*(1, 48) = 3.887, *p* = 0.054), a main effect for TARGET VISIBILITY (*F*(1, 48) = 50.138, *p* < 0.001) but no interaction, i.e., higher PSS in the eyes closed condition (Figure 3A). Romberg’s ratio during HEAD SHAKING did not differ between groups (*p* > 0.8).

Comparing HEAD SHAKING to baseline (head erect, parallel stance) there was a main effect for GROUP (*F*(1,48) = 5.242, *p* = 0.026), TARGET VISIBILITY (*F*(1,48) = 72.111, *p* = 0.001) as well as for the interactions TARGET VISIBILITY × GROUP (*F*(1,48) = 4.335, *p* = 0.043) and HEAD SHAKING × TARGET VISIBILITY (*F*(1,48) = 4.303, *p* = 0.043) but no triple interaction HEAD SHAKING × TARGET VISIBILITY × GROUP (*F*(1,48) = 0.023, *p* > 0.8). Romberg’s ratio during HEAD SHAKING did not change to baseline condition (*F*(1,48) = 3.164, *p* = 0.082) and revealed no group-related differences (*F*(1,48) = 1.920, *p* = 0.172). In summary, postural unsteadiness during head shaking did not differ between groups.

### Dual Task

Analyzing PSS during DUAL TASK, there was a main effect of TARGET VISIBILITY (*F*(1,48) = 32.827, *p* < 0.001) but no main effect of GROUP (*p* > 0.15) and no interaction of GROUP × TARGET VISIBILITY (*p* > 0.08), showing higher PSS for eyes closed condition (Figure 3B). Romberg’s ratio did not differ between groups (*p* > 0.13).

Compared to baseline there was no main effect for the DUAL TASK condition (*F*(1,48) = 0.105, *p* = 0.747) and no GROUP difference (*F*(1,48) = 4.002, *p* > 0.051) but larger PSS during eye closure [TARGET VISIBILITY (*F*(1,48) = 59.567, *p* = 0.001)]. There were interactions of TARGET VISIBILITY × DUAL TASK (*F*(1,48) = 12.765, *p* = 0.001) and TARGET VISIBILITY × GROUP (*F*(1,48) = 5.30, *p* = 0.026) but no DUAL TASK × GROUP interaction (*F*(1,48) = 1.843, *p* = 0.181). There was a main effect for Romberg’s ratio in GROUPs during DUAL TASK (*F*(1,48) = 4.783, *p* = 0.034) with larger ratios for BVF. Furthermore, Romberg’s ratio was lower for DUAL TASK condition (*F*(1,48) = 16.759, *p* < 0.001) while there was no interaction DUAL TASK × GROUP (*p* > 0.32). All in all, cognitive distraction in the dual task paradigm did not dissociate postural performance of patients and controls.

### Proprioceptive Deprivation (Foam)

**Figure 4:**
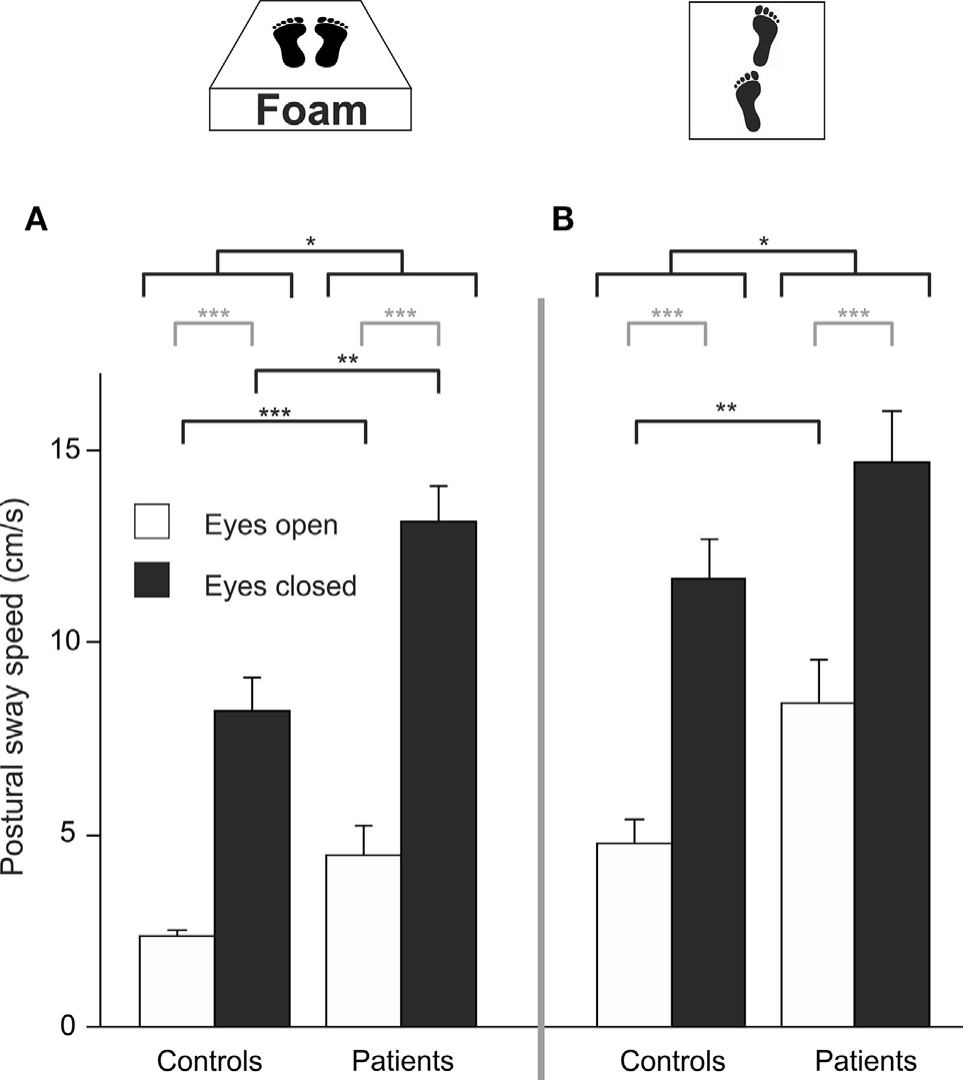
Despite visual control patients show larger PSS on proprioceptive attenuation [foam **(A)**] and tandem stance **(B)**; gray lines indicate within-subject differences, black lines indicate between-subject differences; *p < 0.05, **p < 0.01, ***p < 0.001.

Four patients required postural assistance and were excluded from this analysis. Analyzing PSS during sensory deprivation by foam, there were main effects of GROUP (*F*(1,42) = 19.023, *p* < 0.001) and of TARGET VISIBILITY (*F*(1,42) = 133.218, *p* < 0.001) but no interaction—showing higher PSS for patients and for eyes closed condition (Figure 4A). Romberg’s ratio during FOAM did not differ between groups (*p* > 0.6). Comparing PSS to baseline, there was a main effect of the FOAM paradigm (*F*(1,42) = 138.025, *p* < 0.001) and of TARGET VISIBILITY (*F*(1,42) = 169.573, *p* < 0.001), with GROUP differences (*F*(1, 42) = 14.278, *p* < 0.001), significant interactions for TARGET VISIBILITY × FOAM (*F*(1,42) = 34.104, *p* < 0.001), for FOAM × GROUP (*F*(1,42) = 14.654, *p* < 0.001) and for TARGET VISIBILITY × GROUP (*F*(1,42) = 4.661, *p* < 0.037). Romberg’s ratio on FOAM showed no significant interaction FOAM × GROUP (*p* = 0.135) and no main effects of FOAM (*p* > 0.076) or group differences (*p* > 0.39). With the eyes open, patients showed larger PSS compared to HC (*T*(42) = −3.454, *p* = 0.003). Alltogether, postural sway of patients increased during proprioceptive deprivation by foam compared to controls, with additional significant enlargements in the patients in the absence of visual control (target visibility) on postural stability.

### Tandem Stance

Eight patients required postural assistance and were excluded from this analysis. Analyzing PSS during tandem stance, there were main effects of GROUP (*F*(1, 37) = 6.164, *p* = 0.016) and of TARGET VISIBILITY (*F*(1,37) = 128.554, *p* < 0.001) but no interaction—showing higher PSS for patients and for the eyes closed condition (Figure 4B). Romberg’s ratio during tandem stance was higher for HC than for patients (*T*(36) = 2.141, *p* = 0.039). Compared to baseline, there was a main effect of TANDEM STANCE (*F*(1,37) = 164.119, *p* < 0.001), GROUP (*F*(1,37) = 5.149, *p* = 0.029) and TARGET VISIBILITY (*F*(1,37) = 169.792, *p* < 0.001), an interaction for GROUP × TANDEM STANCE (*F*(1,37) = 7.022, *p* = 0.012), an interaction for TARGET VISIBILITY × TANDEM STANCE (*F*(1,37) = 32.609, *p* < 0.001) but no triple interaction for GROUP × TANDEM STANCE × TARGET VISIBILITY (*p* > 0.2). For Romberg’s ratio, there was no interaction of TANDEM STANCE × GROUP (*p* > 0.12) and no main effects of GROUP (*p* > 0.42) and TANDEM STANCE (*p* > 0.27). Thus, patients showed larger postural instability on tandem stance than controls, irrespective of visual control (target visibility).

### Postural Sway As a Predictor for Vestibular Hypofunction

**Figure 5:**
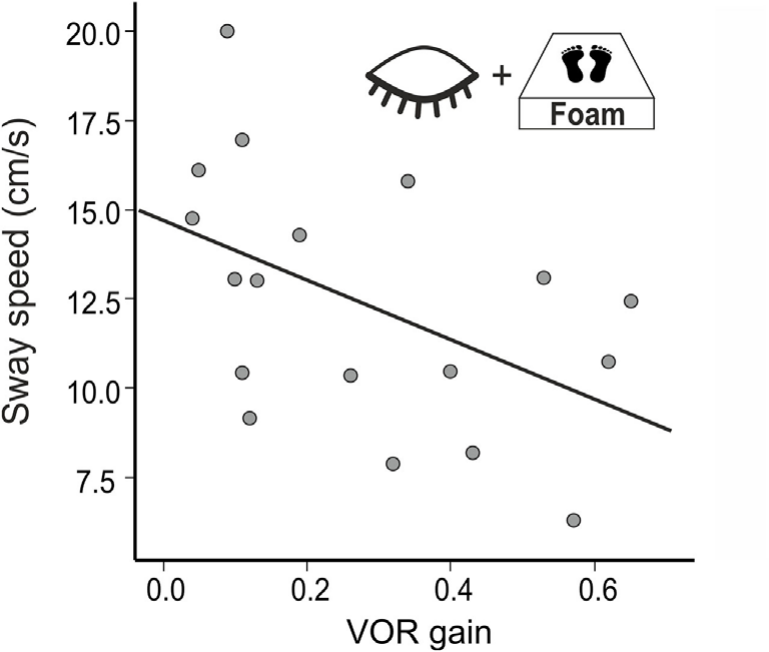
Postural sway speed increases with the severity of vestibular impairment (VOR gain).

In a multiple regression using all conditions postural sway (PSS) explained 70% of the variance of the VOR gain (*R*^2^ = 0.704, *F*(14,38) = 4.085, *p* = 0.001). The best—and the only significant—predictor for vestibular hypofunction (VOR gain) was the standing on foam condition with the eyes closed (*R*^2^ = 0.358, *F*(1,38) = 20.642; *p* < 0.001). Accordingly, PSS of BVF patients increased in the foam paradigm on eye closure with the severity of vestibular impairment (VOR gain reduction, *n* = 18, *r* = −0.486, *p* = 0.041) (Figure 5) but not with disease duration (*p* > 0.055). In none of the experimental conditions, PSS (*p* = 0.557) or Romberg’s ratio (*p* = 0.558) correlated with disease duration.

## Discussion

Sensory control of stable body posture is maintained by error signals deriving from the vestibular, visual, and proprioceptive system (44). They need to be processed, integrated, and weighted as a function of individual demand which may change in disease. Our main findings in our BVF patients were as follows: (1) postural control in BVF using visual and proprioceptive feedback was indistinguishable from HCs. (2) Without visual control BVF, patients consistently showed increased postural sway. (3) Romberg’s ratio at baseline standing condition was larger in BVF. (4) Gravity-dependent tonic head positions in the pitch plane increased postural sway in both groups but the increase in postural sway was larger in BVF on eye closure. (5) Postural unsteadiness during head shaking tended to be larger in patients. (6) Weakening proprioceptive feedback (foam) on postural control heavily increased postural sway in BVF, independent of visual control. Combined proprioceptive and visual deprivation increased postural unsteadiness. (7) Postural control during attentional distraction by the dual task condition did not differ between the groups. (8) Tandem stance heavily destabilized BVF patients.

In comparison to previous studies on the postural control in BVF with proprioceptive and/or visual suppression (22, 45) this study sheds new light on the question how BVF patients stabilize stance when vestibular otolith (head tilt) or SSC stimuli (head shaking) or cognitive distraction tasks are applied during postural control. This constitutes the experimental ground for suggestions for vestibular rehabilitation recommending a decrease of the over-dependence on surface somatosensory inputs by increasing the use of remaining vestibular input (46).

### Visual Control on Posture

From a clinical point of view, it is important to realize that postural control in BVF was indistinguishable from HCs as long as patients can use proprioceptive and visual feedback. Postural control of our BVF patients heavily depended on visual feedback as they showed a strong increase of postural sway on eye closure in all (even in the baseline) conditions compared to the age-matched HCs. This is in line with previous studies (22, 46–50). This increase is reflected by Romberg’s ratio which is used as an indicator of visual and proprioceptive contribution to postural stability (42). In the baseline condition, it was larger in BVF. This dissociates postural control in BVF from patients with vestibulo-cerebellar disorders, e.g., downbeat nystagmus whose increase in postural sway on eye closure (Romberg’s ratio) does not differ from HCs (39). Thus, postural behavior in postural ataxia in degenerative vestibulo-cerebellar disorders and BVF can be distinguished based on (i) baseline standing and (ii) Romberg’s ratio.

### Vestibulo-Spinal Control of Posture

#### Head Tilts

Head tilts in gravity-dependent positions in the pitch plane significantly increased postural sway in both groups. Head tilts activate both otolith (“head-in-space”) signals and proprioceptive neck (“head-on-trunk”) afferents. Both signals are used to calculate the position of the trunk relative to earth-based coordinates such as the line of gravity [“trunk-in-space” (51)]. Vestibulopathic subjects are thought to estimate an erroneous trunk position (trunk-in-space) leading to postural imbalance (52).

The gravity-dependent increase in sway was found in both groups with visual feedback indicating that (i) impaired otolith signal processing in chronic BVF patients has little impact on postural control once the eyes are open and (ii) other factors might counterbalance otolith input to balance control. For example, increased gain in processing of afferent neck proprioceptive signals could substitute reduced/missing otolith contribution in stabilizing posture during head tilt. This intersensory shift could reflect one mechanism of vestibular compensation (24, 53). Another example could be visually mediated perception of body’s posture [e.g., shifted subjective postural or body vertical (54)]. Vision can recalibrate the vestibular reafference signal used to reestablish postural equilibrium (55). Without visual feedback, however, head off-vertical axis weakened postural control in BVF suggesting that deficient otolith signals (reduced ocular vestibular-evoked myogenic potentials) cannot be used sufficiently to stabilize posture. In both groups, Romberg’s ratio was largest in the standard head erect position, which is probably related to the larger sway of BVF in the gravity-dependent head positions at baseline with the eyes open, resulting in a smaller increase on eye closure.

#### Head Shaking

Head shaking modulates horizontal SSC input to vestibulo-spinal control of posture. It also activates proprioceptive neck afferents. Based on the assumption that postural control relies on visual information during head shaking we suspected that head shaking may lead to larger postural sway in BVF due to impaired gaze stabilization. In both groups postural sway increased with head shaking. With the eyes open, postural control in BVF patients did not differ from HCs, despite reduced VOR gain. This is in line with monkeys suffering from mild vestibular ablation which also showed no increase (in fact even a decrease) in postural sway during quiet stance (56) and horizontal head shaking (57). This has been explained by increased muscle-co-contraction (“stiffness”), using a head-fixed-to-foretrunk strategy (57, 58). However, our patients had incomplete but severely reduced VOR gain. Vestibular hypofunction disturbs head-movement related visual acuity in the light. This dynamic visual acuity gets smaller with decreasing VOR gain, at least with passive head movements (59). As our patients were severely impaired on both sides dynamic visual acuity should have been impaired. On a first glimpse, this could imply that visual contribution to postural control during head shaking in our patients is small, despite increased dependence of postural control on visual feedback in BVF (22, 60). However, our BVF patients performed active head movements during head shaking which may result in much smaller impairment and is possibly related to central compensation (61). In fact, 46% of BVF patients had normal dynamic visual acuity during active VOR which may be related to central pre-programming of eye movements or the use of efference copy signals during predictive head movements (62). This may explain why head shaking in our BVF patients had only little impact on postural control. It may have been different if we used passive head movements unpredictable in direction and velocity. This is in contrast to recent animal studies in monkeys suffering from severe bilateral vestibulopathy which showed an increased postural sway during active horizontal head shaking which could be reversed by prosthetic electrical stimulation that partially restored head velocity information (57). Alternatively, active head shaking might have also elicited anticipatory postural adjustments that prevented increased postural sway in BVF (63, 64).

### Proprioceptive Control of Posture

Weakening proprioceptive feedback on postural control by standing on foam showed much stronger postural imbalance (PSS) in BVF compared to controls, even with visual feedback support. This is in line with the enhanced proprioceptive dependence of postural control in chronic BVF (22, 65) and the destabilizing effect of additional diseases affecting proprioceptive feedback control on posture, e.g., in polyneuropathy (66). In combined deprivation of visual and proprioceptive feedback signals (foam condition with the eyes closed), some BVF patients required short postural assistance and needed to be excluded. This explains the high sensitivity (80%) of the “Romberg’s test on foam rubber” in BVF (67) as it provokes a stronger dependence of postural control on vestibular input. In healthy subjects, normal VOR is sufficient to maintain balance under these multisensory deprivations but BVF patients fall off the mattress if VOR is heavily impaired. Accordingly, there was an increase of postural sway the stronger VOR gain was reduced (Figure 5). Therefore, patients with severe BVF should be informed about increasing postural unsteadiness and risk of falls when they lack firm support beneath their feet or suffer from additional polyneuropathy.

### Dual Task Effects on Posture

Dual postural-cognitive task conditions have been used to study the relationship between attention and postural control. This relation is highly age-dependent (68): older subjects have higher attentional demands for postural control and show slower reaction times during combined postural-cognitive task (69). This leads to a higher risk of falling during standing and walking while talking (70). Our BVF patients could maintain postural control during attentional distraction in the dual task condition indistinguishable from age-matched HCs as long as visual and proprioceptive feedback was assured. This distinguishes BVF patients from the elderly (71), cerebellar patients (72) or patients with phobic postural vertigo (PPV) (73). The increased and inadequate use of sensory feedback in PPV patients suspected to cause their postural imbalance normalizes by distracting cognitive tasks (74, 75). This is not the case in BVF patients who largely rely on closed-loop mechanisms of postural control. This dependence on proprioceptive feedback may probably be even stronger as they showed a higher Romberg’s ratio in the dual task condition compared to controls. Unfortunately, severity of postural imbalance of our BVF patients did not allow us to investigate whether they maintain stance under more challenging dual task conditions (e.g., on foam).

### Increased Motor Demand on Postural Balance (Tandem Stance)

Increased multisensory and motor postural demands (tandem stance) heavily destabilized BVF patients. Postural control of BVF patients was highly impaired compared to HCs, even when visual and proprioceptive input is used. Additional visual deprivation elicited a stronger postural imbalance compared to the HC group. Tandem stance requires multisensory integration, including vestibular input as visual and proprioceptive feedback is not sufficient to stabilize stance in BVF. This is in line with the concept of vestibular compensation in which postural control in vestibular failure is compensated by improving the sensory weight of unaffected sensory systems (24), i.e., they rely stronger on visual and proprioceptive feedback sources to maintain postural control (60). Accordingly, patients with uni-sensory deficit have a smaller risk of falling that patients with impairment of multiple sensory inputs required for postural control (66). It remains to be investigated whether the increased risk of falls in BVF (66) is related to increased co-contractions of antagonistic muscle groups as found in patients with cerebellar disease (72) and PPV (75).

### Limitations of the Study

Individual BVF patients may vary in the extent they exercise vestibular rehabilitation and accordingly they may vary in the magnitude of vestibular compensation. At the time of recording, vestibular compensatory mechanisms should have been established with respect to the average disease duration of our patients (3.1 years), if they developed at all. Therefore, we cannot specify how individual exercise influenced the variability of postural sway but can only refer to the group effects.

## Conclusions

In conclusion, diagnosis of BVF patients is often missed possibly because postural control in BVF at baseline is indistinguishable from HCs once visual and proprioceptive input is provided. In comparison with cerebellar DBN patients, BVF patients show a stronger visual dependency (increase in Romberg’s ratio). The best postural predictor for BVF is the condition with standing on foam with the eyes closed. Accordingly, our data suggest that BVF should be tested with the eyes closed while standing on foam (mattress test). The strong dependency of postural control in BVF on proprioceptive and visual cues should be taken into consideration in vestibular rehabilitation.

## Ethics Statement

The study protocol was approved by the institutional Ethics Committee of the University of Lübeck (project ID: 11-119; February 10, 2012). It was in accord with the ethical guidelines listed in the declaration of Helsinki and its subsequent amendments. This study was carried out in accordance with the recommendations of the Ethics Committee of the University of Luebeck (https://www.uni-luebeck.de/forschung/kommissionen/ethikkommission/sonstige-studien.html) with written informed consent from all subjects. All subjects gave written informed consent in accordance with the Declaration of Helsinki. The protocol was approved by the Ethics Committee of the University of Luebeck.

## Author Contributions

AS contributed to study design, methodology, statistical analysis, critical reviewing, and editing of the manuscript. JFW and NMJ contributed to methodology, data acquisition, statistical analysis, and reviewing of manuscript. CH contributed to conceptualization and study design, project administration, data acquisition, supervision, drafting, editing, and approving the final writing of the manuscript.

## Conflict of Interest Statement

The authors declare that the research was conducted in the absence of any commercial or financial relationships that could be construed as a potential conflict of interest.
